# Ablation of the Proapoptotic Genes Chop or Ask1 Does Not Prevent or Delay Loss of Visual Function in a P23H Transgenic Mouse Model of Retinitis Pigmentosa

**DOI:** 10.1371/journal.pone.0083871

**Published:** 2014-02-11

**Authors:** Adeseye Adekeye, Mohammad Haeri, Eduardo Solessio, Barry E. Knox

**Affiliations:** Departments of Neuroscience & Physiology, Biochemistry & Molecular Biology and Ophthalmology, Center for Vision Research, SUNY Upstate Medical University, Syracuse, New York, United States of America; National Eye Institute, United States of America

## Abstract

The P23H mutation in rhodopsin (Rho^P23H^) is a prevalent cause of autosomal dominant retinitis pigmentosa. We examined the role of the ER stress proteins, Chop and Ask1, in regulating the death of rod photoreceptors in a mouse line harboring the Rho^P23H^ rhodopsin transgene (*GHL^+^*). We used knockout mice models to determine whether Chop and Ask1 regulate rod survival or retinal degeneration. Electrophysiological recordings showed similar retinal responses and sensitivities for *GHL^+^*, *GHL^+^/Chop^−/−^* and *GHL^+^/Ask1^−/−^* animals between 4–28 weeks, by which time all three mouse lines exhibited severe loss of retinal function. Histologically, ablation of *Chop* and *Ask1* did not rescue photoreceptor loss in young animals. However, in older mice, a regional protective effect was observed in the central retina of *GHL^+^/Chop^−/−^* and *GHL^+^/Ask1^−/−^*, a region that was severely degenerated in *GHL^+^* mice. Our results show that in the presence of the Rho^P23H^ transgene, the rate of decline in retinal sensitivity is similar in *Chop* or *Ask1* ablated and wild-type retinas, suggesting that these proteins do not play a major role during the acute phase of photoreceptor loss in *GHL^+^* mice. Instead they may be involved in regulating secondary pathological responses such as inflammation that are upregulated during later stages of disease progression.

## Introduction

Retinitis pigmentosa (RP) is a group of inherited retinal diseases that are characterized by the degeneration of the retina. RP has an incidence rate of ∼1 in 4000 [1] and can be classified based on the mode of inheritance [2], [3]. To date, mutations in over 50 genes have been associated with RP [4]. Mutations in rhodopsin (Rho), the visual pigment of rod photoreceptors, accounts for ∼10% of all cases of RP [1]. These mutations have been grouped on the associated functional defects and cellular distribution of the protein [5]. In North America, the class II missense mutation P23H (Rho^P23H^), is the most common and accounts for >25% of all autosomal dominant forms of RP [4]. This mutation leads to a misfolded protein that is characterized by abnormal glycosylation pattern, abnormal disulfide bond formation and oligomerization [6]. In RP patients with the Rho^P23H^ allele, retinal degeneration is characterized by the initial disorganization and shortening of rod outer segments, followed by the loss of rod photoreceptors and the ensuing death of cone cells [7], [8]. Similar findings are observed in various Rho^P23H^ animal models [9]–[12]. However, the cellular mechanisms that lead to cell death are not completely understood.

Since Rho^P23H^ is a misfolded protein, recent studies have examined the up-regulation of endoplasmic reticulum stress (ER-stress)/unfolded protein response (UPR) pathways with retinal degeneration [13]–[15]. The UPR is an intracellular response to perturbations of ER homeostasis. It is regulated by the Hsp70 family protein chaperone, BiP [16]. Under normal conditions, BiP binds to three ER transmembrane proteins, ATF6, IRE1, and PERK. In a stressed state, BiP dissociates, and ATF6, IRE1 and PERK pathways are activated, resulting in the upregulation of protective events to decrease ER stress (reviewed in [17]). However, failure to restore ER homeostasis results in the upregulation of cell death pathways [18]–[21]. Two genes, C/EBP homologous protein-10 (Chop) and apoptosis signal-regulating kinase 1 (Ask1), have been identified as important regulators of UPR induced cell death/apoptosis [22]–[26].

Chop is a transcription factor [27] that is regulated mainly by the PERK branch of the UPR response and has a functional role in the other pro-apoptotic pathways of the UPR [25], [28], [29]. Ask1 is a kinase activated by the IRE1 division of the UPR pathway [30]–[32]. It activates downstream kinases that induce apoptosis, and post-translationally phosphorylates Chop to positively modify its activity [33]. Several studies have shown that altering the expression of UPR genes, including Chop and Ask1, can change the pathology of different diseases [22]–[24], [26], [34]–[36]. The role of the UPR in RP is not completely understood. Although CHOP was elevated in cells transfected with Rho^P23H^ [14], it is not clear whether that also occurs in rod photoreceptors. In transgenic rats expressing Rho^P23H^, expression of Chop and BiP were increased in degenerated retinas [13]–[15]. Moreover, overexpression of BiP can restore some visual function in this animal [13]. Upregulation of Ask1 has also been linked to retinal degenerative diseases [37]. More recently, the Ask1 pathway has been implicated as a regulator of photoreceptor death in a transgenic RP mouse model expressing a Rho^T17M^ mutation [38]. Taken together, these findings suggest that UPR genes may regulate photoreceptor death in RP, and regulating the ER stress response might be beneficial to the treatment of RP. However, direct evidence is lacking that establishes the upregulation of UPR proteins as a necessary response for photoreceptor death.

To determine whether Chop or Ask1 regulate photoreceptor cell death, we utilized knockout mice and a well characterized Rho^P23H^ transgenic mouse model (*GHL^+^*) [9]. Similar to human patients, RP in *GHL^+^* mice is characterized by the progressive decline in visual function, and morphological changes in the retina that include shortening of rod outer segments and thinning of the outer nuclei layer due to death of photoreceptor cells [9], [39]. The disease pathology of *GHL^+^* mice is also comparable to recently generated Rho^P23H/+^ heterozygote knock-in mice [11], which also show a slow progressive degeneration of the retina characterized by shortening of rods followed by death of rod photoreceptors. We assessed the role of Chop and Ask1 proteins as possible modifiers of *GHL^+^* induced pathology by generating *GHL^+^*/*Chop^−/−^* and *GHL^+^*/*Ask1^−/−^* mice and characterized the progression of retinal degeneration electrophysiologically and histologically over a 7-month period. We observed that ablation of either of these genes did not affect the rate of photoreceptor death in early stages of the disease, however a regional protective effect was observed in the central retina at later stages of the disease. In *GHL^+^* mice, the central retina degenerates faster than more peripheral regions of the retina. Therefore, our findings suggest that deleting Chop and Ask1 may have an indirect role in protecting the retina by modifying late stage responses to ongoing photoreceptor death, resulting in a slight delay of photoreceptor death.

## Materials and Methods

### Animal models

All animal handling and experiments were in agreement with the animal care and use guidelines of the Association for Research in Vision and Ophthalmology (ARVO). This study was done under the approval of the SUNY Upstate Medical University Committee on the Human Use of Animals (CHUA no. 182 and 323). C57BL/6 and *Chop^−/−^* animals were purchased from Taconic and the Jackson Laboratory respectively. *Ask1^−/−^* mice were obtained from H. Ichijo (University of Tokyo, Japan). *GHL* ^+^ transgenic mice [9] were obtained from C. Cepko (Harvard Medical School, Boston, MA). The *GHL^+^* mice express a transgene encoding a mutated mouse opsin gene containing three amino acid substitutions at the N-terminus: V20G, P23H, and P27L (*GHL^+^* mice). Mice were housed in a 14 h light–10 h dark cycle.

### Genotyping

Genomic DNA from tail snips were isolated either by boiling in NaOH or using the Phire® Animal Tissue Direct PCR Kit (Fisher Scientific Pittsburgh, PA) according to manufacturer's instructions. The genotype of each gene was confirmed by PCR: *Chop*, [40], *GHL^+^* [9], *Ask1^−/−^* [32], SOX21 (Phire® Animal Tissue Direct PCR Kit) and β-actin (Promega, Madison, WI) were used as loading controls.

### Electroretinogram (ERG) recordings

ERG recordings were performed as described by [41]–[43] with some modifications. Animals were dark adapted overnight in a light-proof cage and prepared for ERG recordings under dim red light and infrared (IR) illuminators. Animals were anesthetized with an i.p. injection of 90 mg/kg Nembutal. The pupil of the stimulated eye (right eye) was dilated with 1% Tropicamide, and corneal moisture of the non-stimulated eye (left eye) was maintained with 0.3%glycerin/1% propylene glycerol eye drops or Gonak™ Hypromellose Solution, 2.5%. The mice were placed on a heating pad in a light-tight Faraday cage. Reference and ground electrodes were placed in the mouth and subcutaneously in the tail, respectively. A Burian-Allen electrode (Hansen Ophthalmic Development Laboratory, Coralville, IA) was placed on the corneal surface of the right eye. Following electrode placement under IR illumination, animals were dark adapted for 7–10 minutes prior to presentation of the first light stimulus. The response signal was amplified, band-pass filtered (0.3 Hz and 100 Hz) and digitized at 1 kHz. The light stimulus consisted of a series of 1 ms LED flashes (λ = 520 nm) presented in increments from −3.25 log cds/m^2^ to +3.17 log cds/m^2^. +3.17 log cds/m^2^ = 5.76 log photoisomerizations/rod (log Rh*/rod). To reduce the effects of noise, records for −3.25, −2.25, and −1.25 log cds/m^2^ are the average of 9 stimulus presentations presented at 30 second intervals, stimulus for −0.25, +0.74, +1.74, and +3.17 logcds/m^2^ were presented 4 times at 2 minute intervals. The a-wave was measured from the baseline to the trough of the initial negative wave, while the b-wave was measured from the trough of the a-wave to the peak of the initial positive wave. All responses were recorded with Clampex and analyzed using Clampfit 10.0 software (Molecular Devices LLC, Sunnyvale, CA). Averaged data was analyzed for graphical presentation with SigmaPlot 11.0 (Systat Software Inc., San Jose, CA). ERG recordings were repeated every 28–31 days over a 7-month period. The numbers of animals used for each experiment are presented in the figure legends.

### Data analysis

Statistical analysis was performed using Student's *t-test* with SigmaPlot 11.0 software. All values are expressed as ± standard deviation (SD). The amplitudes of a- and b-wave intensity response function plots were fitted with Hill function (n = 0.8). Threshold intensities (*I_t_*) required to evoke criteria a- and b-wave amplitudes of 50 µV and 75 µV respectively were extrapolated from Hill function fits of a- and b-wave intensity response function plots.

### Histology

Eyes were enucleated and placed in a fixative mixture of 2% glutaraldehyde/4% paraformaldehyde in PB followed by embedding in JB-4 embedding media according to manufacturer's protocol (JB-4 embedding kit, Polysciences, Inc. Warrington, PA), sectioned at 2 µm thickness using the Sorvall MT-5000 Ultra Microtome, and stained with 1% (w/v) toluidine blue stain (Ricca Chemical Company, Arlington, TX). Changes in retinal morphology were determined by counting the number of nuclei per column in the outer nuclear layer (ONL). To account for the non-uniform degenerative changes in the retina, the total number of nuclei per column represents averaged nuclei counts from multiple regions, superior, central, and inferior zones, over a distance of 100 µm. The superior and central zones were in the dorsal portion of the retina and were measured ∼600 µm from the ciliary marginal zone and optic nerve respectively. The inferior zone was measured 1.1 mm from the CMZ in the ventral portion of the retina.

### Quantitative Real time PCR (qRT-PCR)

Retinas were separated from the RPE and surrounding eye tissues, quickly frozen on dry ice and stored at −80°C until use. Total RNA was prepared using the RNeasy kit (Qiagen). RNA integrity was assessed using the Agilent 2100 bioanalyzer. mRNA was reverse transcribed using the Quantitect Reverse Transcription kit (Qiagen). PCR reaction was prepared using the LightCycler ® 480 SYBR Green I Master (Roche). Primers used were obtained from the PrimerBank database (http://pga.mgh.harvard.edu/primerbank/). PCR thermal cycles were as follows: *heat activation*: 95°C for 10 min, *amplification* (42 cycles): 95°C for 15 s, 60°C for 15 s, 72°C for 15 s, 80°C for 5 s, and *melt curve*: 95°C for 10 s, 45°C for 1 min with a heating rate of 0.1°C. The threshold cycle crossing point (Cp) of the gene of interest (GOI) in each sample was normalized to the Cp of *β-actin* of the same sample [44]. The derived normalized Cp value for each animal was then averaged to get the mean normalized Cp for each genotype and was used to define the relative expression of each gene.

## Results

### Effects of Chop on photoreceptor sensitivity

**Figure 1:**
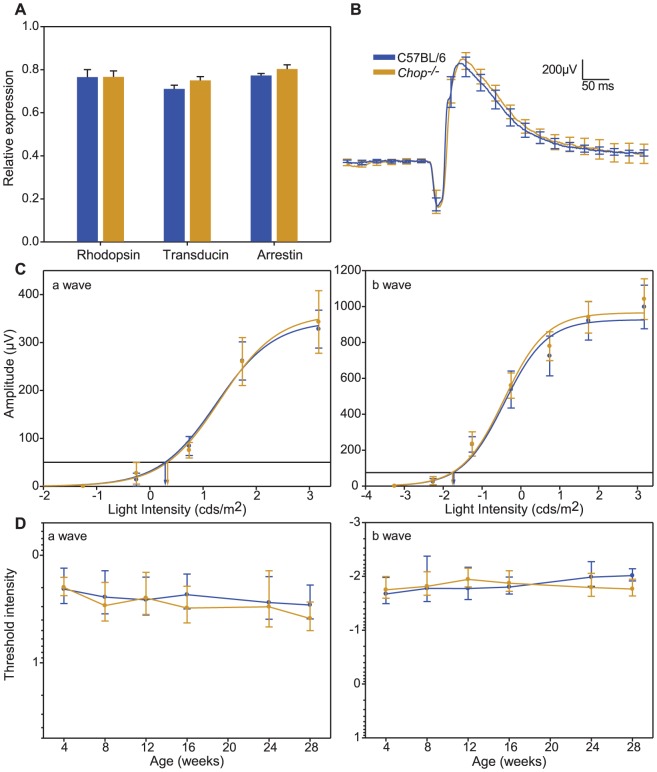
Effect of *Chop* on the expression of phototransduction genes and visual function. (A). Quantitative RT-PCR analysis of 4 week old C57BL/6 and *Chop^−/−^* mice (*n = 4*). Samples were normalized to β-actin levels. (B). Average scotopic ERG a- and b-wave waveforms from 4 week old C57BL/6 (blue trace) and *Chop^−/−^* (orange trace) mice in response to 1 ms flashes (n = 7–9). (C) Intensity-response functions of a- and b-waves recorded from the same C57BL/6 and *Chop^−/−^* mice. Horizontal lines and vertical intercepts on the abscissa (a-wave *I_t_*: 50 µV, b-wave *I_t_*: 75 µV) show the method used to determine threshold light intensities (*I_t_*). (D) Age related changes in a- and b-wave threshold light intensities in C57BL/6 and *Chop^−/−^* mice. Error bars are ± SD.

Since Chop has been shown to regulate the expression of rhodopsin through a microRNA, miR-708, in 293T cells [45], deleting Chop could alter rhodopsin levels in photoreceptors and potentially influence retinal degeneration. Therefore, we analyzed mRNA levels of rhodopsin, arrestin and transducin using RT-PCR analysis on RNA from 4 week old *Chop* ^−/−^ and C57BL/6 mice (Fig. 1A). There was no significant difference in the normalized mean expression levels of rhodopsin between *Chop^−/−^* and C57BL/6 mice (*p = 0.962, n = 4*) and there was less than a 5% difference in arrestin (*p = 0.033, n = 4*) and transducin (*p = 0.021, n = 4*) mRNA levels. Therefore in mouse photoreceptors, Chop does not appear to regulate the expression of rhodopsin and minimally affects the expression of transducin and arrestin.

**Figure 2:**
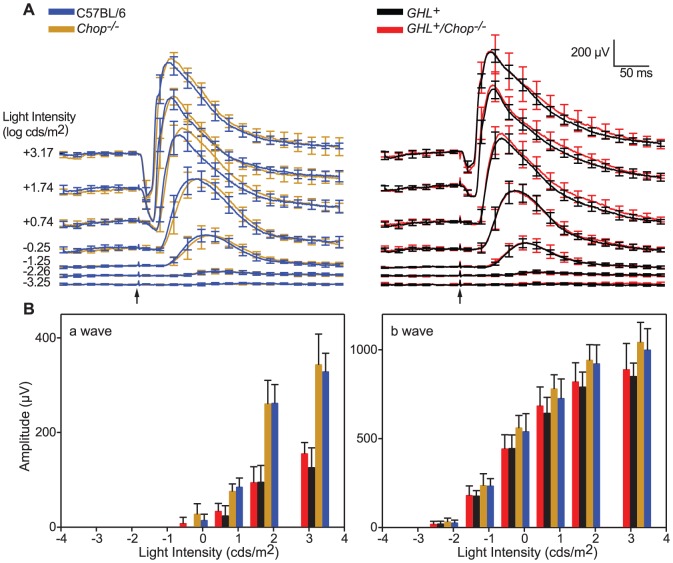
ERG responses in 4 week old *GHL^+^* and *GHL^+^/Chop^−/−^* mice. (A) Average scotopic ERG a- and b-wave waveforms from C57BL/6 (blue traces), *Chop^−/−^* (orange traces) *GHL^+^* (black traces) and *GHL^+^/Chop^−/−^* (red traces) mice in response to 1 ms flashes of increasing intensity, from bottom to top, (*n = 7–9*). (B) Intensity-response functions of a- and b-waves. Error bars: ± SD. Scale bar: x = 50 ms, y = 200 µV. Arrows represent the onset of light stimulus.

To characterize the retinal sensitivity in *Chop^−/−^* mice, we used electrophysiology to record scotopic ERG waveforms as a function of light intensities. Representative ERG traces recorded in response to a saturating flash (Fig. 1B) and to a series of flashes covering the range of intensities used in this study (Fig. 2A) are shown. The cornea-negative leading edge of the ERG waveform, the a-wave, originates from photoreceptor cells [46], and the maximal positive potential gives the b-wave amplitude and represents responses by second order bipolar cells [47]–[49]. The intensity-response curves for both a-wave (Fig. 1C) and b-wave (Fig. 1C) from *Chop^−/−^* and C57BL/6 animals were indistinguishable at 4 weeks.

We characterized changes in visual sensitivity by determining the light intensity, defined as the threshold intensity (*I_t_*), required to evoke neural responses at criteria a- and b-wave amplitudes of 50 µV and 75 µV respectively over a 7-month period (Fig. 1D), which falls in the linear range of the response of the retinal cells to the presented stimulus. At 4 weeks, the mean a-wave was comparable between C57BL/6 and *Chop^−/−^* mice (*p = 0.820*, Table 1). Over the 7-month period, the a-wave *I*_t_ was similar in C57BL/6 and *Chop^−/−^* mice (*p>0.05*) (Table 1). Similarly, over the 7-month period there was no significant difference between the b-wave *I*_t_ in C57BL/6 and *Chop^−/−^* mice (*p = 0.461*, Table 2). Since we did not observe differences in a-wave *I*_t_ between C57BL/6 and *Chop^−/−^* mice, the results show that photoreceptor sensitivity is not altered by ablation of *Chop*.

**Table 1:** a-wave threshold intensity (I_t_) values.

Age (weeks)						
	4	8	12	16	24	28
a-wave I_t_ (cds/m^2^)						
**C57**	2.1±0.7	2.4±1.1	2.6±1.0	2.3±0.8	2.8±1.2	2.9±1.0
***Chop^−/−^***	2.0±0.4	2.9±1.1	2.5±1.1	3.1±1.2	3.0±1.6	3.9±1.1
***Ask1^−/−^***	2.3±1.3	3.4±1.0	3.6±2.7	2.7±0.9	2.8±0.7	5.1±2.4
***GHL^+^***	26.7±25.6	25.6±14.6	38.3±34.5	61.6±46.3	162±109	ND
***GHL^+^*** **/** ***Chop^−/−^***	18.0±13.5	37.5±31.5	59.1±66.3	163±137	68.1±50.9	ND
***GHL^+^/Ask1^−/−^***	12.2±5.0	17.0±11.0	44.2±28.4	95.7±80.9	68.8±44.7	ND

Threshold amplitude: 50 µV, I_t_: Threshold intensity, ND: not detectable.

**Table 2:** b-wave threshold intensity (I_t_) values.

Age (weeks)						
	4	8	12	16	24	28
b-wave I_t_ (mcds/m^2^)						
**C57**	21.3±10.8	16.8±12.6	16.8±10.1	15.8±5.5	10.3±5.0	9.7±2.5
***Chop^−/−^***	17.8±7.7	15.3±7.1	11.4±4.3	13.4±5.6	16.1±7.4	17.2±5.9
***Ask1^−/−^***	35.2±52.1	12.6±6.3	18.0±15.0	19.7±18.2	19.6±11.6	32.2±16.1
***GHL^+^***	28.5±12.4	39.2±25.1	33.7±14.6	128±142	482±1190	252±313
***GHL^+^/Chop^−/−^***	35.5±30.8	31.1±16.5	33.4±17.6	80.7±55.3	142±179	281±232
***GHL^+^/Ask1^−/−^***	18.8±7.3	16.8±7.8	26.2±9.5	72.9±79.8	90.5±55.3	146±167

Threshold amplitude: 75 µV, I_t_: Threshold intensity.

### Effect of Chop on *GHL^+^* retinal degeneration at 4 weeks

*GHL^+^* transgenic mice exhibit a progressive loss in a-wave and b-wave amplitudes over time compared to non-transgenic animals [9]. We compared the retinal sensitivity of *GHL^+^* and *GHL^+^/Chop^−/−^* transgenic mice by measuring scotopic ERG responses (Fig. 2A) over a range of light intensities. Representative ERG waveforms show that the a- and b-wave responses increased progressively with increasing light intensity (Fig. 2A). We used responses at maximal amplitudes (*saturating light intensity*) and criteria a- and b-wave amplitudes (*threshold intensity* = *I_t_*, *see above*) to characterize physiological changes in the retina. We defined differences in retinal function between the genotypes as biologically relevant if statistical significance was observed for maximal amplitude and threshold intensity measurements.

At the saturating light intensity, there was a ∼62% reduction in the a-wave amplitude of 4 week old *GHL^+^* (*p<0.001*) compared to C57BL/6 and *Chop^−/−^* mice (Fig. 2B). Similarly, threshold intensities decreased by ∼13 fold in 4 week old *GHL^+^* compared to age matched control mice (Table 1). The b-wave amplitude of *GHL^+^* mice at saturating light intensity decreased by ∼15% (*p<0.01*) compared to control mice, the threshold intensity (*I_t_*) was higher than control mice, however the difference was not statistically significant when compared to in C57BL/6 mice (*p = 0.242*).

In 4 week old *GHL^+^/Chop^−/−^* mice, there was a ∼53% reduction in the a-wave amplitude at the saturating light intensity compared to C57BL/6 and *Chop^−/−^* mice (*p<0.001*). This was 24% higher than *GHL^+^* mice at the same light intensity (Fig. 2B, *see above*), but the difference was not statistically significant (*p = 0.126, n = 7–9*). Also, a-wave *I*_t_ was significantly different from C57BL/6 and *Chop^−/−^* mice (*p = 0.009*), but not significantly different compared to *GHL^+^* mice (*p = 0.424*).

4 week old *GHL^+^/Chop^−/−^* mice had an ∼11% reduction in b-wave amplitude of compared to C57BL/6 (*p = 0.153*) and *Chop^−/−^* mice (*p = 0.033*). The maximum b-wave amplitudes of *GHL^+^/Chop^−/−^* and *GHL^+^* mice were not statistically different (*p = 0.515*) (Fig. 2B). Similarly, the b-wave *I*_t_ was not significantly different from *GHL^+^* mice (*p = 0.541*) (Table 2).

**Figure 3:**
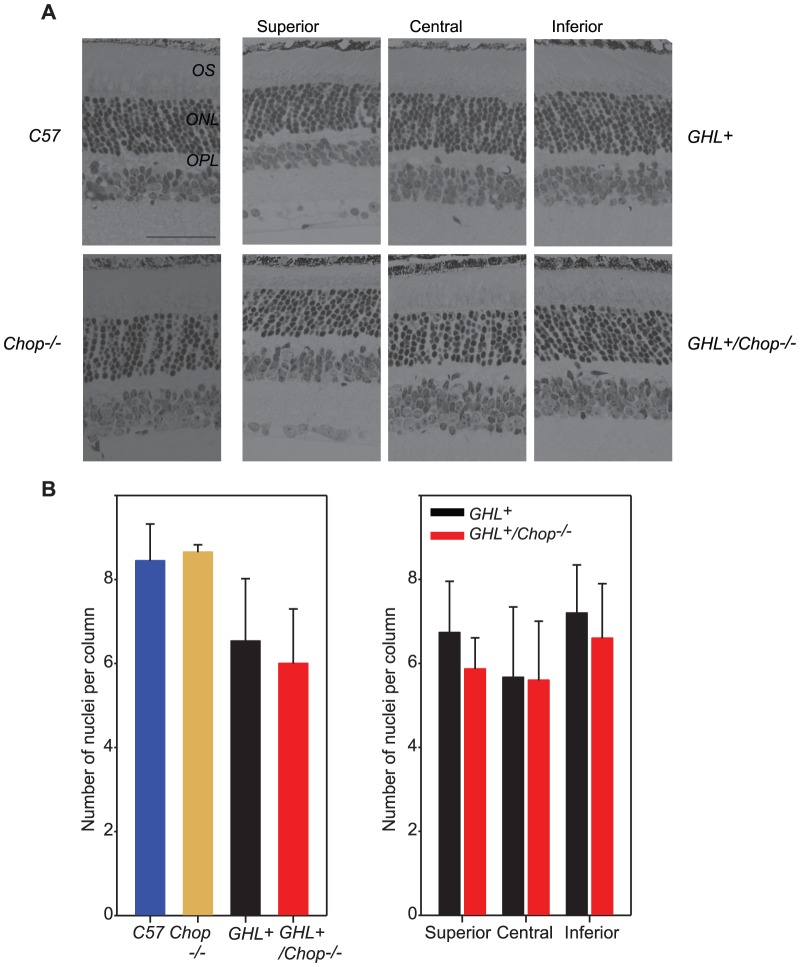
Histological analysis of 4 week old *GHL^+^* and *GHL^+^/Chop^−/−^* retinas. (A) Representative sections of retinas from 4 week old C57BL/6, *Chop^−/−^*, *GHL^+^* and *GHL^+^/Chop^−/−^* mice (*n = 2–3*). (B) Mean number of photoreceptor nuclei per column counted in the ONL (*left panel*), and the variance in the number of photoreceptor nuclei per column counted in different zones of the retina in *GHL^+^* and *GHL^+^/Chop^−/−^* mice, (*right panel*). Superior zone: 630 µm from the CMZ, central zone: 630 µm from optic nerve, and inferior zone: midpoint of inferior hemisphere. Error bars: ± SD OPL: outer plexiform layer, ONL: outer nuclear layer, OS: outer segment. Scale bar, 50 µm.

We determined the number of photoreceptor nuclei across the retina at 4 weeks. In C57BL/6 and *Chop^−/−^* mice, the ONL had 8.6±1.5 and 8.6±1.2 nuclei per column respectively (Fig. 3). In *GHL^+^* mice, the overall architecture of the retina was similar to control mice, however there was a decrease in the number of photoreceptor nuclei in the ONL. To account for the possibility of variation in the extent of photoreceptor loss across the retina, we divided the whole retina into three different zones, the superior zone, the central zone and the inferior zone (Fig. 3). In 4 week old *GHL^+^* mice (*n = 3*), the number of photoreceptor nuclei per column in superior zone was 6.7±1.2. There were 5.7±1.7 in the central zone and 7.2±1.1 nuclei per column in the inferior zone, representing an overall average of 6.5±1.5 nuclei per column. On average this represents a ∼24% reduction in the number of photoreceptors compared to control (*p<0.001*) animals. The reduction in the number of photoreceptor nuclei in the ONL and shortening of the OS [9], [51] are consistent with observed reductions in the a-wave amplitude of *GHL^+^* mice.

In 4 week old *GHL^+^/Chop^−/−^* mice (*n = 3*), the number of photoreceptor nuclei in superior zone was 5.9±0.7 per column. There were 5.6±1.4 in the central zone and 6.6±1.3 in the inferior zone, representing an overall average of 6.0±1.3. Accordingly, there was no significant difference between the mean number of photoreceptors in *GHL^+^/Chop^−/−^* and *GHL^+^* mice (*p = 0.086*) (Fig. 3). Therefore, our results demonstrate that the reduction in a-wave and b-wave maximal amplitudes, threshold intensities, and the number of remaining photoreceptors in 4 week old *GHL^+^*- expressing mice appears to be independent of expression of Chop.

### Effect of Chop on *GHL^+^* retinal degeneration at 16 weeks

**Figure 4:**
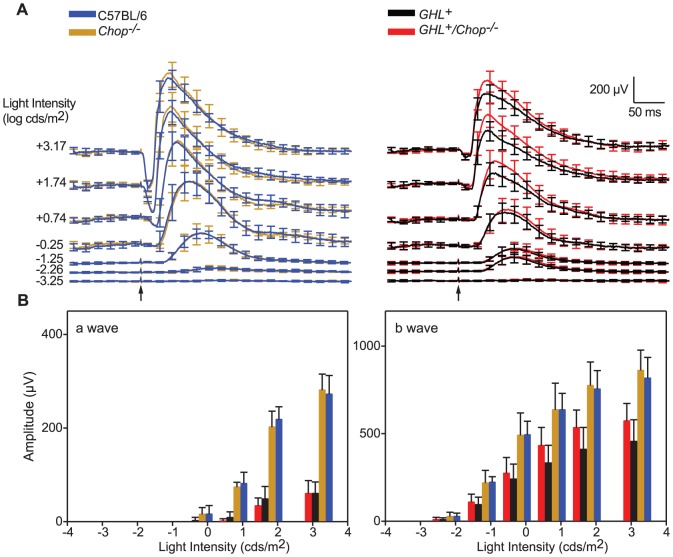
ERG responses in 16 week old *GHL^+^* and *GHL^+^/Chop^−/−^* mice. (A) Average scotopic ERG a- and b-wave waveforms from C57BL/6 (blue traces), *Chop^−/−^* (orange traces) *GHL^+^* (black traces) and *GHL^+^/Chop^−/−^* (red traces) mice in response to 1 ms flashes of increasing intensity, from bottom to top, (*n = 8–13*). (B) Intensity-response functions of a- and b-waves. Error bars ± SD. Scale bar: x = 50 ms, y = 200 µV. Arrows represent the onset of light stimulus.

At 16 weeks, the approximate midpoint in retinal degeneration in *GHL^+^* mice [51], the maximum a-wave amplitude at saturating light intensity decreased by 52% compared to 4 week old *GHL^+^* mice (Fig. 4). The a-wave amplitude was 22% of C57BL/6 and *Chop^−/−^* mice. In some animals the a-wave could not be detected at intensities <+1.74 log cds/m^2^. The a-wave *I*_t_ decreased by ∼20 fold compared to control mice (*p<0.01*). At the saturating light intensity, there was a ∼44% decline in the b-wave amplitude of *GHL^+^* mice compared to age matched control animals (*p<0.001*). The b-wave *I*_t_ was significantly different compared to C57BL/6 mice (*p = 0.041*) (Table 2).

In 16 week old *GHL^+^/Chop^−/−^* mice, the maximum a-wave amplitude at saturating light intensity decreased by 61% compared to 4 week old mice. The a-wave amplitude was 22% (*p<0.001*) that of 16 week old C57BL/6 and *Chop^−/−^* mice. There was no significant difference between maximum a-wave amplitudes in *GHL^+^/Chop^−/−^* and *GHL^+^* mice (*p = 0.994*) (Fig. 4). In 16 week old *GHL^+^/Chop^−/−^* mice, a-wave *I*_t_ was significantly different compared to C57BL/6 and *Chop^−/−^* mice (*p<0.01*), but not significantly different from *GHL^+^* mice (*p = 0.089*) (Table 1).

At the saturating light intensity, there was a ∼30% reduction in the b-wave amplitude of 16 week old *GHL^+^/Chop^−/−^* mice compared to age matched control mice. The maximum b-wave in *GHL^+^/Chop^−/−^* mice was ∼25% higher compared to *GHL^+^* mice (*p = 0.018*) (Fig. 4B). In 16 week old *GHL^+^/Chop^−/−^* mice, the b-wave *I*_t_ was not significantly different compared to *GHL^+^* mice (*p = 0.279*).

**Figure 5:**
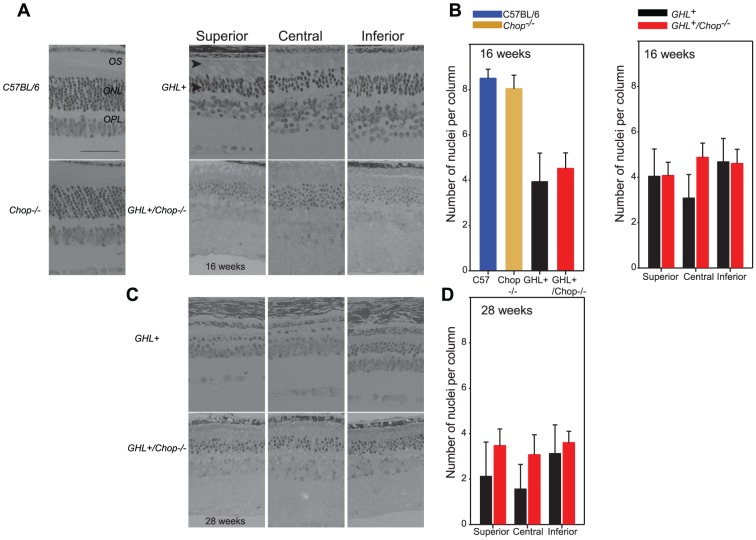
Histological analysis of 16 and 28 week old *GHL^+^* and *GHL^+^/Chop^−/−^* mice. (A) Representative sections of retinas from 16 week old C57BL/6, *Chop^−/−^*, *GHL^+^* and *GHL^+^/Chop^−/−^* mice. *GHL^+^* and *GHL^+^/Chop^−/−^* retinas had shortened OS (arrowheads). (B, *right panel*) Mean number of photoreceptor nuclei per column counted in the ONL at 16 weeks. The variance in the number of photoreceptor nuclei per column counted in different zones of the retina in (B, *left panel*) in 16 week. (C) Representative sections of retinas from 28 week old *GHL^+^* and *GHL^+^/Chop^−/−^* mice. (D) The variance in the number of photoreceptor nuclei per column counted in different zones of the retina in 28 week old *GHL^+^* and *GHL^+^/Chop^−/−^* mice. Superior zone: 630 µm from the CMZ, central zone: 630 µm from optic nerve, and inferior zone: midpoint of inferior hemisphere. Error bars: ± SD OPL: outer plexiform layer, ONL: outer nuclear layer, OS: outer segment. Scale bar 50 µm.

We determined the number of photoreceptor nuclei across the retina at 16 weeks. In C57BL/6 and *Chop^−/−^* mice, the ONL had 8.5±1.7 and 8.4±1.5 nuclei per column, respectively (Fig. 5A). By this time, retinas of *GHL^+^* mice were severely degenerated [9], with shortened OS and a disorganized ONL with far fewer nuclei per column (Figure 5B). In 16 week old *GHL^+^* mice (*n = 5*), the number of photoreceptor nuclei in superior zone was 4.0±1.2. There were 3.1±1.0 in the central zone and 4.7±1.0 in the inferior zone (Fig. 5A, B). On average the total number of photoreceptor nuclei per column in the ONL was 3.9±1.3.

In 16 week old *GHL^+^/Chop^−/−^* mice (*n = 3*), the number of photoreceptor nuclei in superior zone was 4.1±0.6 per column. There were 4.9±0.6 nuclei per column in the central zone and 4.6±0.6 in the inferior zone, representing an overall average of 4.5±0.7 nuclei per column (Fig. 5A, B). Unlike *GHL^+^* mice, the pattern of degeneration in *GHL^+^/Chop^−/−^* mice was more uniform across the retina. When we compared the nuclei count in the different zones between *GHL^+^/Chop^−/−^* and *GHL^+^* mice, the degree of degeneration in the central zone was significantly higher in *GHL^+^* animals than in *GHL^+^/Chop^−/−^* mice (*p<0.001*), while the differences in the superior and inferior zones were more comparable with *p values* of *0.937* and *0.788* respectively (Fig. 5B). This, results suggest Chop may influence late stage responses that occur as the disease progresses.

### Age related changes in retinal degeneration of *GHL^+^/Chop^−/−^* mice

**Figure 6:**
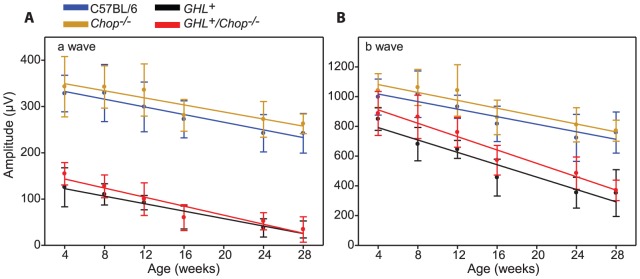
ERG changes as a function of age in *GHL^+^* and *GHL^+^/Chop^−/−^* mice. Maximal a- and b-wave amplitudes as a function of age in C57BL/6 (blue trace), *Chop^−/−^* (orange trace) *GHL^+^* (black trace) and *GHL^+^/Chop^−/−^* (red trace) mice. Colored lines are polynomial fits.

We determined the effect of Chop on the rate of degeneration during the major phase of rod loss, between 4–28 weeks. In control C57BL6 and *Chop^−/−^* mice there were ∼26% (*p = 0.001*) and ∼23% (*p = 0.008*) losses in maximum a-wave amplitudes, respectively, which is similar to what has been reported previously [50] (Fig. 6A). Linear regression for a-wave amplitudes between 4 and 28 weeks showed a downward trend with age in C57BL/6 mice (−4.15±0.51 µV/week) and *Chop^−/−^* mice (−3.8±0.73 µV/week) (Fig. 6A). Likewise, there were 24% (*p = 0.004*) and ∼26% (*p<0.001*) losses in maximum b-wave amplitudes respectively, in C57BL6 and *Chop^−/−^* mice (Fig. 6B, Fig. S1). Linear regression of b-wave amplitudes trended downwards in C57BL/6 mice (−12.7±2.3 µV/week) and *Chop^−/−^* mice (−13.3±2.5 µV/week) (Fig. 6B). Therefore the rate of loss of retinal responses was similar in C57BL/6 and *Chop^−/−^* mice.

In *GHL^+^* mice, both maximal a-wave and b-wave amplitudes were severely reduced at all time points compared to control animals. The maximal a-wave amplitude, while significantly lower than control animals (*see above*) at 4 weeks, continued to decline so that by 28 weeks it was only 27% (*p<0.001*) of the initial 4 week value (and ∼13% that of 28 week old C57BL/6 and *Chop^−/−^* mice) (Fig. 6A). In a few *GHL^+^* animals, the a-wave was no longer detectable at 28 weeks. Regression of a-wave amplitudes with age was −4.04±0.43 µV/week in *GHL^+^* mice (Fig. 6A). The maximal b-wave amplitude declined by ∼60% (*p<0.001*) between 4 and 28 weeks in *GHL^+^* mice, and was ∼45% (*p<0.001*) that of C57BL/6 and *Chop^−/−^* mice (Fig. 6B). Regression of b-wave amplitudes with age was −20.8±3.04 µV/week in *GHL^+^* mice (Fig. 6B).

In *GHL^+^/Chop^−/−^* mice, both maximal a-wave and b-wave amplitudes were also severely reduced at all time points compared to control animals. The maximal a-wave amplitude declined to 22% (*p<0.001*) of the 4 week value (and ∼13% that of 28 week old C57BL/6 and *Chop^−/−^* mice). Regression of a-wave amplitudes with age was −4.9±0.72 µV/week in *GHL^+^/Chop^−/−^* mice (Fig. 6A). The rate of decline in a-wave responses was similar in C57BL/6, *Chop^−/−^*, *GHL^+^* and *GHL^+^/Chop^−/−^* mice, however, there were significant reductions in maximal a-wave amplitudes and higher threshold intensities (*I_t_*) in *GHL^+^* and *GHL^+^/Chop^−/−^* mice compared to control mice (*see above*). The maximal a-wave amplitudes and threshold intensities were not significantly different between *GHL^+^* and *GHL^+^/Chop^−/−^* mice, suggesting similar ongoing changes in the loss of photoreceptor cells and decrease in rhodopsin concentrations in the *GHL^+^* and *GHL^+^/Chop^−/−^* mice retinas.

The maximal b-wave amplitude declined by 58% (*p<0.001*) of the 4 week value in *GHL^+^/Chop^−/−^* mice (Fig. 6B). Between 8–24 weeks, the maximum b-wave amplitudes were 15–27% higher (*p<0.05*) in *GHL^+^/Chop^−/−^* compared to *GHL^+^* mice. However, at 4 and 28 weeks there was no statistical difference in between *GHL^+^/Chop^−/−^* and *GHL^+^* b-wave maximal amplitudes. Regression of b-wave amplitudes with age was −22.5±2.23 µV/week in *GHL^+^/Chop^−/−^* mice (Fig. 6B). The rate of decline in b-wave responses was similar in *GHL^+^* and *GHL^+^/Chop^−/−^*, and faster than C57BL/6, *Chop^−/−^* control mice. Thus, the overall loss of visual sensitivity in *GHL^+^/Chop^−/−^* and *GHL^+^* mice declined at similar rates.

**Figure 7:**
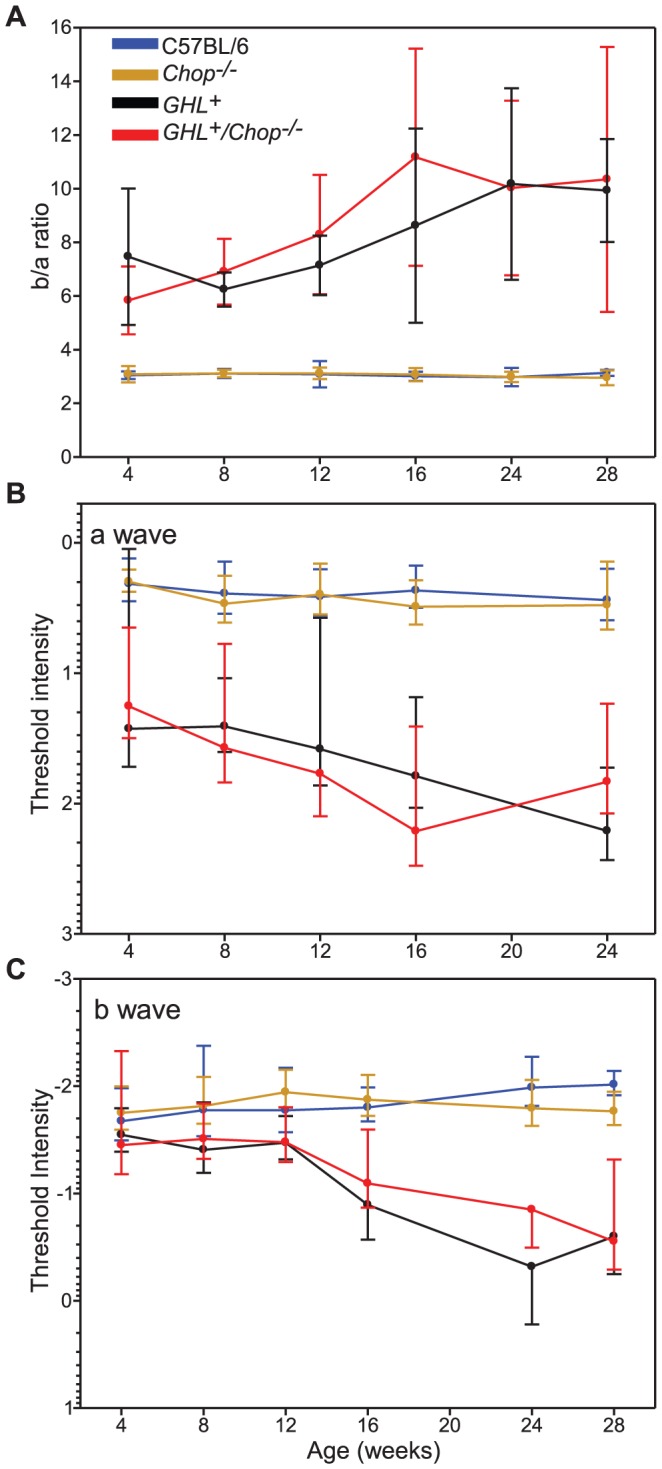
Age related changes in retinal function and sensitivity of *GHL^+^* and *GHL^+^/Chop^−/−^* mice. (A) b/a wave ratio in C57BL/6, *Chop^−/−^*, *GHL^+^*, and *GHL^+^/Chop^−/−^* mice. (B and C) Age related changes in a- and b-wave threshold intensities in C57BL/6, *Chop^−/−^*, *GHL^+^*, and *GHL^+^/Chop^−/−^* mice (a- wave *I_t_* = 50 µV, b- wave *I_t_* = 75 µV). Error bars: ± SD.

Since the b-wave was higher in *GHL^+^/Chop^−/−^* compared to *GHL^+^* mice, we assessed the signal transmission from photoreceptor cells to second order cells by determining the b/a wave ratio [52]. We determined the b/a ratio by normalizing maximal b-wave amplitudes to maximal a-wave amplitudes and plotted the values as a function of age (Fig. 7A). The b/a ratio is constant in healthy retinas, therefore, deviations from normal b/a ratio represent pathological/degenerative changes in the retina [52]. The b/a ratio was similar between C57BL/6 and *Chop^−/−^* mice (Fig. 7A). In *GHL^+^* mice, the b/a ratio was abnormally high at all ages compared to control mice, which is consistent with ongoing degeneration. Similarly, in *GHL^+^/Chop^−/−^* mice, the b/a ratio was higher than C57BL/6 and *Chop^−/−^* animals. At all ages, b/a ratio was comparable between *GHL^+^/Chop^−/−^* and *GHL^+^* mice (*p>0.05*), suggesting that there are no significant differences in signal transmission from photoreceptors to second order cells in *GHL^+^/Chop^−/−^* and *GHL^+^* mice.

In addition, we determined the number of photoreceptor nuclei across the retina at 28 weeks. Since no significant differences were observed in the pattern of survival of photoreceptor cells between C57BL/6 and *Chop^−/−^* mice, at 28 weeks we focused on the degenerative changes between *GHL^+^* and *GHL^+^/Chop^−/−^* mice. By 28 weeks, retinas of *GHL^+^* mice (*n = 5*) were almost completely degenerated [9], with absent OS and the number of nuclei per column ranged from zero to four (Fig. 5C). The number of photoreceptor nuclei in superior zone was 2.1±1.5. There were 1.6±1.1 in the central zone and 3.1±1.3 in the inferior zone. Similar to the pattern observed at 16 weeks, the central zone was more degenerated than the more peripheral superior and inferior zones (Fig. 5C, D).

In 28 week old *GHL^+^/Chop^−/−^* mice (*n = 3*), the number of photoreceptor nuclei per column in superior zone was 3.5±0.7, there were 3.1±0.9 nuclei per column in the central zone, and 3.6±0.5 in the inferior zone (Fig. 5C, D). Again, the retina continued to uniformly degenerate in different zones of the retina, which was consistent with the pattern we observed at 16 weeks. When we compared the nuclei count in the different zones between *GHL^+^/Chop^−/−^* and *GHL^+^* mice, the degree of degeneration in the central zone was significantly higher in *GHL^+^* animals compared to *GHL^+^/Chop^−/−^* mice (*p<0.001*). In addition, the degree of degeneration in the superior zone was also significantly higher in *GHL^+^* animals than *GHL^+^/Chop^−/−^* mice (*p = 0.003*) (Fig. 5D). However, inferior zones were more comparable with a *p value* of *0.171* (Fig. 5D). These results suggest that although Chop does not directly prevent photoreceptor death, it may influence late stage responses of surrounding cells and/or other ongoing cellular responses in the retina to dying photoreceptors and as such can prolong cell survival in older animals.

### Effect of Ask1 on *GHL^+^* retinal degeneration at 4 weeks

**Figure 8:**
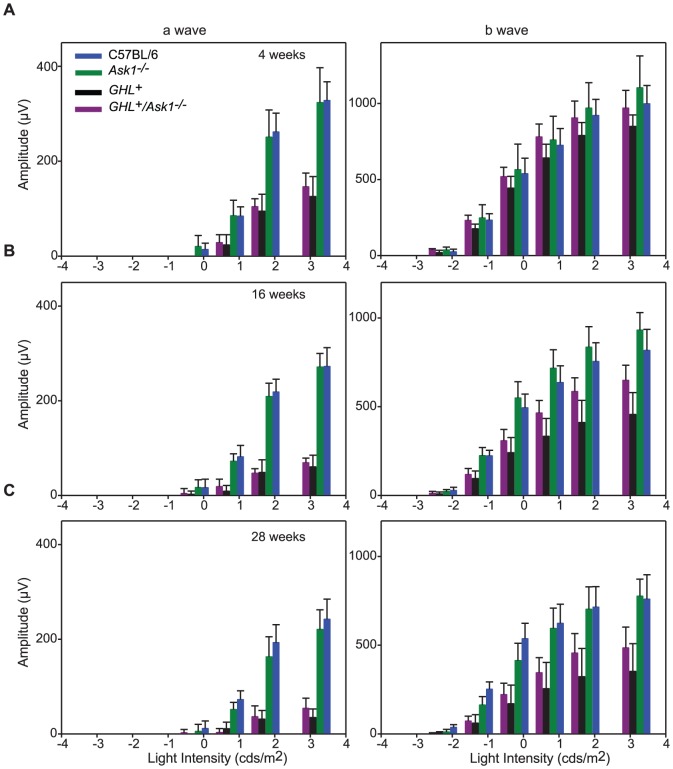
ERG responses in *GHL^+^* and *GHL^+^/Ask1^−/−^* mice. Intensity-response functions of a- and b-waves in (A) 4, (B) 16, and (C) 28 week old C57BL/6 (blue), *Ask1^−/−^* (green) *GHL^+^* (black) and *GHL^+^/Ask1^−/−^* (purple) mice (*n = 7–14*). Error bars: ± SD.

At the saturating light intensity, there was a ∼55% decreased in the a-wave amplitude of 4 week old *GHL^+^/Ask1^−/−^* mice compared to C57BL/6 and *Ask1^−/−^* mice (*p<0.001*). The maximum a-wave amplitude was 17% higher than the maximum a-wave of *GHL^+^* mice (Fig. 8A), however the difference was not statistically significant (*p = 0.294, n = 7–9*). Similarly, the a-wave *I*_t_ (Table 1) was significantly different from C57BL/6 and *Ask1^−/−^* mice (*p<0.001*), but not significantly different from *GHL^+^* mice (*p = 0.163*).

At the saturating light intensity, the b-wave decreased by 3% and 12% compared to C57BL/6 (*p = 0.663*) and *Ask1^−/−^* mice (*p = 0.162*), respectively. The maximum b-wave amplitudes of *GHL^+^/Ask1^−/−^* and *GHL^+^* mice were statistically different (*p = 0.027*) (Fig. 8A). The b-wave *I*_t_ was not statistically different from *GHL^+^* mice (*p = 0.089*) (Table 2).

### Effect of Ask1 on *GHL^+^* retinal degeneration at 16 weeks

In 16 week old *GHL^+^/Ask1^−/−^* mice, the maximum a-wave amplitude at the saturating light intensity decreased by 53% compared to 4 week old animals. The a-wave amplitude was 25% that of C57BL/6 and *Ask1^−/−^* mice (*p<0.001*). There was no significant difference between maximum a-wave amplitudes in *GHL^+^/Ask1^−/−^* and *GHL^+^* mice (*p = 0.360*) (Fig. 8B). The a-wave *I*_t_ was significantly different from C57BL/6 and *Ask1^−/−^* mice (*p<0.01*), but not significantly different from *GHL^+^* mice (*p = 0.345*) (Table 1).

At the saturating light intensity, there was a ∼25% decrease in b-wave amplitudes of 16 week old *GHL^+^/Ask1^−/−^* mice compared to C57BL/6 and *Ask1^−/−^* mice. The maximum b-wave in *GHL^+^/Ask1^−/−^* mice was ∼42% higher compared to *GHL^+^* mice (*p = 0.002*) (Fig. 8B). The b-wave *I*_t_ was not statistically different from *GHL^+^* mice (*p = 0.338*) (Table 2).

### Age related changes in retinal degeneration of *GHL^+^/Ask1^−/−^* mice

**Figure 9:**
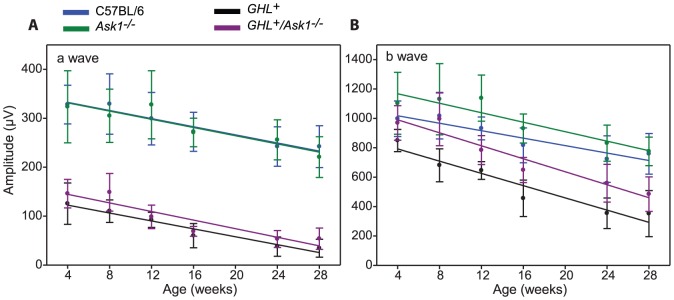
Rate of retinal degeneration in *GHL^+^* and *GHL^+^/Ask1^−/−^* mice. Maximal a- and b-wave amplitudes as a function of age in C57BL/6 (blue), *Ask1^−/−^* (green) *GHL^+^* (black) and *GHL^+^/Ask1^−/−^* (purple) mice. Error bars: ± SD. Colored lines are polynomial fits.

We determined the effect of Ask1 on the rate of degeneration during the major phase of rod loss, between 4–28 weeks. In control *Ask1^−/−^*, mice there was a ∼30% (*p = 0.003*) age related loss in maximum a-wave amplitudes, (Fig. 9A, Fig. S2). Linear regression for a-wave amplitudes between 4 and 28 weeks showed a downward trend with age in *Ask1^−/−^* mice (−4.2±0.87 µV/week) (Fig. 9A). Likewise, there was a 30% (*p<0.001*) age related loss in maximum b-wave amplitude. Linear regression of b-wave amplitudes trended downwards in *Ask1^−/−^* mice (−16.1±3.13 µV/week) (Fig. 9B).

In *GHL^+^/Ask1^−/−^* mice, both maximal a-wave and b-wave amplitudes were severely reduced at all time points compared to control animals. At 28 weeks, the maximal a-wave amplitude was 54±22 µV, which declined to 37% (*p<0.001*) of the 4 week value (and ∼22% that of C57BL/6 and *Ask1^−/−^* mice at 28 weeks, *p<0.001*). Regression of a-wave amplitudes with age was −4.4±0.89 µV/week in *GHL^+^/Ask1^−/−^* mice (Fig. 9A). The rate of decline in a-wave responses was similar in C57BL/6 (*see above*), *Ask1^−/−^*, *GHL^+^* and *GHL^+^/Ask1^−/−^* mice, however, there were significant reductions in maximal a-wave amplitudes compared to control mice. The maximal a-wave amplitudes were not significantly different between *GHL^+^* and *GHL^+^/Ask1^−/−^* mice, suggesting similar ongoing changes with respect to loss of photoreceptor cells and decrease in rhodopsin concentrations.

**Figure 10:**
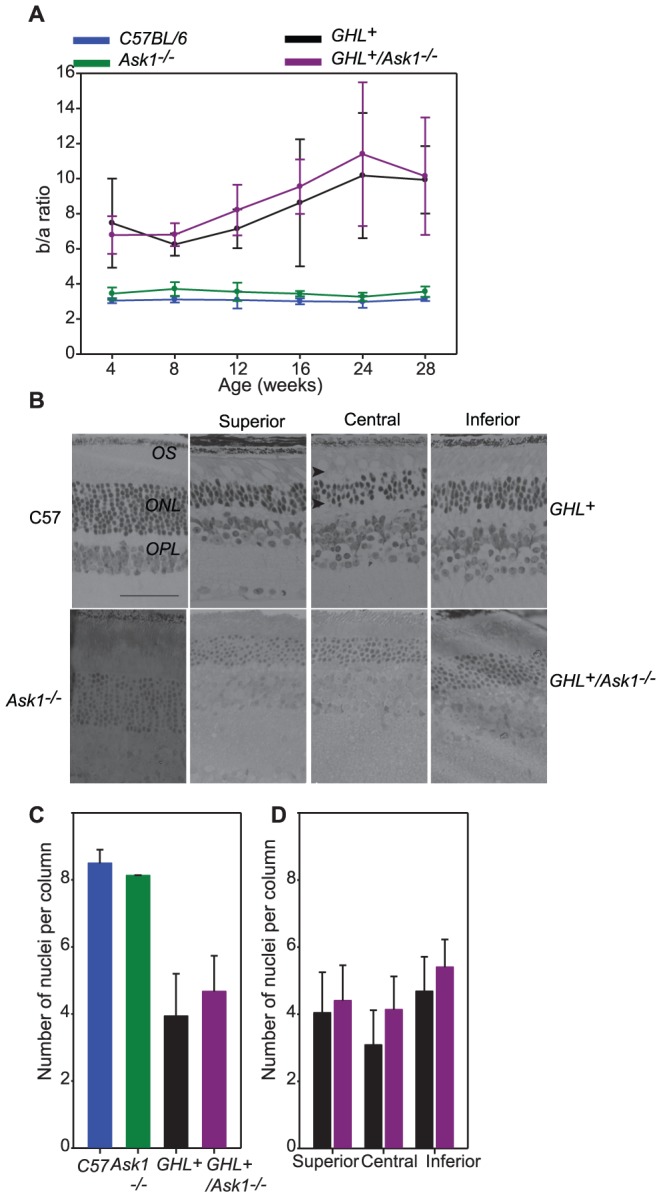
Age related changes in retinal function and morphology of *GHL^+^* and *GHL^+^/Ask1^−/−^* mice. (A) b/a wave ratio in C57BL/6, *Ask1^−/−^*, *GHL^+^*, and *GHL^+^/Ask1^−/−^* mice. (B) Representative sections of retinas from 16 week old C57BL/6, *Ask1^−/−^*, *GHL^+^* and *GHL^+^/Ask1^−/−^* mice. *GHL^+^* and *GHL^+^/Ask1^−/−^* retinas had shortened OS (arrowheads). (C) Mean number of photoreceptor nuclei per column counted in the ONL. Error bars: ± SD. OPL: outer plexiform layer, ONL: outer nuclear layer, OS: outer segment. Scale bar 50 µm. (D) The variance in the number of photoreceptor nuclei per column counted in different zones of the retina in *GHL^+^* and *GHL^+^/Ask1^−/−^* mice, (*right panel*). Superior zone: 630 µm from the CMZ, central zone: 630 µm from optic nerve, and inferior zone: midpoint of inferior hemisphere.

At 28 weeks, the maximal b-wave amplitude (484±117 µV) declined by 50% (*p<0.001*) of the 4 weeks value in *GHL^+^/Ask1^−/−^* mice (Fig. 9B). Between 4–28 weeks, the maximum b-wave amplitudes were 14–57% higher (*p<0.01*) in *GHL^+^/Ask1^−/−^* compared to *GHL^+^* mice. Regression of b-wave amplitudes with age was −22.1±3.11 µV/week in *GHL^+^/Ask1^−/−^* mice (Fig. 9B). The rate of decline in b-wave responses was similar in *GHL^+^* and *GHL^+^/Ask1^−/−^* but faster than C57BL/6, *Ask1^−/−^* control mice. However, there were significant differences in maximal b-wave amplitudes in *GHL^+^* and *GHL^+^/Ask1^−/−^* mice compared to control mice. To assess signal transmission through the retina we determined the b/a ratio of *GHL^+^/Ask1^−/−^* mice (Fig. 10A). The b/a ratio was similar between C57BL/6 and *Ask1^−/−^* mice (Fig. 10A). In *GHL^+^/Ask1^−/−^* mice, the b/a ratio was higher than control C57BL/6 and *Ask1^−/−^* animals, but was comparable to *GHL^+^* mice (*p>0.05*) (Fig. 10A).

We determined the number of photoreceptor nuclei across the retina at 16 weeks. In *Ask^−/−^* mice, the ONL had 8.1±1.7 nuclei per column (Fig. 10B). In 16 week old *GHL^+^/Ask1^−/−^* mice (*n = 3*), the number of photoreceptor nuclei in superior zone was 4.4±1.1 per column, there were 4.1±1.0 nuclei per column in the central zone, and 5.4±0.8 nuclei per column in the inferior zone, representing an overall average of 4.7±1.1 (Fig. 10B, C). Unlike *GHL^+^* mice, this pattern of degeneration was more uniform across the retina. When we compared the nuclei count in the different zones between *GHL^+^/Ask1^−/−^* and *GHL^+^* mice, the degree of degeneration in the central zone was significantly higher in *GHL^+^* animals than *GHL^+^/Ask1^−/−^* mice (*p = 0.003*), while the differences in the superior and inferior zones were more comparable with *p values* of *0.345* and *0.027* respectively (Fig. 10B, C). This, results suggest that similar to Chop, Ask1 may also influence late stage responses of surrounding cells and/or other cellular responses in the retina to dying photoreceptors and as such can prolong cell survival in older animals.

## Discussion

The purpose of this study was to determine whether Chop or Ask1 regulate photoreceptor death in the Rho^P23H^ transgenic mouse model of RP. If Chop or Ask1 are positive regulators of photoreceptor death, deficiency of these genes should preserve a- and b-wave response amplitudes, threshold intensities, and retinal morphology. On the other hand if Chop or Ask1 are negative regulators of cell death, ablation of these genes should enhance the loss of retinal function and structure. However, ablation of *Chop* or *Ask1* genes did not alter the rate of loss of maximal a-wave amplitudes in *GHL^+^*-expressing mice. Electrophysiological recordings showed that deleting *Chop* or *Ask1* genes did not preserve a-wave amplitudes, threshold intensities (*I_t_*), and the rate of loss of photoreceptor responses in *GHL^+^/Chop^−/−^* and *GHL^+^/Ask1^−/−^* mice from 4 to 28 weeks. At all light intensities, the a-wave amplitude and *I_t_* values were not significantly different between *GHL^+^* and *GHL^+^/Chop^−/−^* or *GHL^+^/Ask1^−/−^* mice. These results imply that Chop and Ask1 do not preserve overall photoreceptor function and sensitivity in *GHL^+^*-expressing mice.

Histological analysis of 4,16, 28 week old *GHL^+^* mice retinas revealed a regional difference in the rate of photoreceptor loss. Central zones of the retina degenerated faster than the more peripheral zones, consistent with other reports using these mice [9]. The superior and central zones are in the dorsal portion of the retina, while the inferior zone is in the ventral portion. One possible explanation for the differences in the rate of loss of photoreceptor cells in different zones of the retina may be attributable to a variance in the expression of the transgene in the dorsal and ventral retina. Since there is no way to differentiate between protein levels of the GHL transgene and endogenous opsin, we cannot confirm this regional effect in transgene expression.

At 4 weeks, the magnitude of decline in the a-wave amplitude was higher compared to the b-wave amplitude between control animals and mice expressing the GHL transgene. A possible explanation is that at this age, the photoreceptor responses can still elicit saturating responses in bipolar cells, which is represented by the smaller decline in b-wave amplitudes in GHL+ expressing animals. While in 16 to 28 week old mice, more photoreceptors have been lost resulting in a larger decline in the generated a-wave amplitudes driving the b-wave responses, which is represented by the larger decline in b-wave amplitudes in GHL+ expressing animals.

The b-wave amplitude was higher in *GHL^+^/Chop^−/−^* and *GHL^+^/Ask1^−/−^* compared to *GHL^+^* mice. At most time points, the increase was significant at the higher light intensities compared to low/medium light intensities. b-wave amplitudes are driven by photoreceptor responses (a-wave) and synaptic connections with bipolar cells. The transfer of signals from photoreceptors to second order cells is non-linear [53]. Therefore changes in synaptic connections, cellular integrity and inter-cellular connections between rods, cones and bipolar cells may alter b-wave amplitudes. One possible explanation for the differences in b-wave amplitudes at the higher intensities is that Chop and Ask1 may have pleiotropic effects on the retina, and the loss of these genes may have influence other processes in inner retinal cells. To determine the physiological effects of deleting *Chop* and *Ask1* genes, we assessed signal transmission from photoreceptors to second order cells by characterizing the b/a ratios [52]. At all ages (4–28 weeks), the b/a ratios were not significantly different between *GHL^+^* and *GHL^+^/Chop^−/−^* or *GHL^+^/Ask1^−/−^* mice. Also, there was no significant difference in the rate of loss of b-wave amplitudes between *GHL^+^* and *GHL^+^/Chop^−/−^* or *GHL^+^/Ask1^−/−^* mice. Therefore, physiological analysis suggests that signal transmission through the retina and number of inner retinal cells was similar in *GHL^+^* and *GHL^+^/Chop^−/−^*, *GHL^+^/Ask1^−/−^* mice at all ages.

When we compared the pattern of degeneration and number of nuclei per column in the ONL between *GHL^+^* and *GHL^+^/Chop^−/−^*, *GHL^+^/Ask1^−/−^* mice, we observed an age dependent difference between the pattern of degeneration and number of surviving cells in the different zones of the retina. There were no significant differences in number of photoreceptor nuclei per column between 4 week old *GHL^+^* and *GHL^+^/Chop^−/−^*, *GHL^+^/Ask1^−/−^* mice. By contrast, in older mice, the central zone was particularly less degenerated in *GHL^+^/Chop^−/−^* and *GHL^+^/Ask1^−/−^* mice compared to *GHL^+^* mice. This was an interesting finding because ERG a-wave amplitudes at 16 and 28 weeks showed no differences in retinal sensitivity between *GHL^+^* and *GHL^+^/Chop^−/−^* or *GHL^+^/Ask1^−/−^* mice. One possible explanation for this is that since ERG recordings represent a summed response of all photoreceptor cells, and differences ∼1–2 row variance in nuclei count between *GHL^+^* and *GHL^+^/Chop^−/−^*, *GHL^+^/Ask1^−/−^* mice, cannot be readily measured by this technique. Therefore, although ablation of Chop and Ask1 do not preserve overall functional response of the retina, the benefits of targeting the UPR in treating RP may be more apparent during later stages of the disease when the difference in the number of photoreceptors remaining is between no cells to one or two rows of cells.

In a recent study by Nashine et al. [54], ablation of Chop did not protect against retinal degeneration over a 3 month period in the Rho^T17M^ transgenic mice RP model. That finding is similar to our results on *GHL^+^*. However, the Rho^T17M^ transgenic mice may have exhibited a cytoprotective effect with Chop in the early stages of retinal degeneration since one month old Rho^T17M^ Chop^−/−^ mice had lower a-wave amplitudes and thinner ONL compared to age matched Rho^T17M^ mice. The authors suggested that Chop deficiency causes a decrease in the rhodopsin expression via transcriptional mechanisms. These findings differ from our results on *GHL^+^/Chop^−/−^* mice, which exhibited some protection from cell death at later stages. Although photoreceptor death is the common fate in both Rho^T17M^ and *GHL^+^* transgenic mice, each model may lead to cell death via different intracellular pathways [55]. Besides the different rhodopsin mutations used, there are also differences in the rhodopsin protein sequence (Rho^T17M^ transgene encodes the human protein while *GHL^+^* transgene encodes the mouse protein). Previous studies using mammalian cell culture showed that there is a species difference in the expression and biochemical properties of Rho^T17M^ [6], [56]. In addition, there are also possible differences in transgene expression or mosaicism between the two models. In fact, overexpression of wild-type opsin transgenes in mouse retinas has been shown to induce retinal degeneration [10]. Thus it is not clear what accounts for the differing effects of Chop on cell death in Rho^T17M^ and *GHL^+^* transgenic mice. To understand whether various rhodopsin mutants cause cell death by similar pathways it will be necessary to compare knock-in lines.

The ablation of Ask1 has a protective effect in different neurodegenerative disease models [24], [57]. In addition, an increase in the expression of Ask1 in the *tubby* mouse model of retinal degeneration suggests a role for Ask1 in other retinal degenerative diseases [37]. Ask1 was recently linked to photoreceptor death in the Rho^T17M^ mice model [38]. This occurs by the Ask1 regulation of the c-Jun N-terminal kinase (c-JNK) pathway via the tumor necrosis factor (TNF)-dependent pathway ultimately regulating caspase-7 [32]. Together, these results suggest that Ask1 may have an indirect role in regulating cell death in Rho^T17M^. Our results are consistent with these findings, however the effects are small and only apparent at later stages. The delayed and partial rescue of photoreceptor cells suggests that Ask1 may influence chronic degenerative changes in the retina by modifying late onset pathological responses like inflammatory pathways.

The involvement of UPR pro-apoptotic genes in many diseases, such as diabetes, Huntington's disease and Parkinson's disease, is well established [22], [24], [26], [34], [35], and Chop and Ask1 have been shown to prolong cell survival in the aforementioned diseases. However, based on our results these branches of the UPR do not regulate the acute loss of photoreceptor cells in transgenic *GHL^+^* mice. In fact, it appears that the influence on photoreceptor death may be through indirect mechanisms of modulating later pathological changes in the retina during disease progression. A possible explanation for the regional effect of deleting Chop and Ask1 is that since older photoreceptors reside in the central retina and are known to die faster than younger peripheral photoreceptors in different RP models [9], [58], deleterious effects associated with the expression of Chop and Ask1 occur earlier in these cells than cells in the retinal periphery. Therefore, accounting for the regional protection of deleting *Chop* and *Ask1* in retinas of older animals.

Morphological changes in the outer segment are one of the earliest findings associated with disease pathology in RP patients. It has been established that the proper formation of the OS is required for the survival of rod photoreceptors [59]–[61]. Thus, it is possible that the observed early onset decrease in a-wave amplitudes in *GHL^+^*, *GHL^+^/Chop^−/−^* and *GHL^+^/Ask1^−/−^* mice is associated with the observed changes in the rod OS structure. The consequences of the presence of Rho^P23H^ in the OS are unknown. Based on recent findings, we showed that Rho^P23H^ forms aggregates that lead to the disruption of discs in the OS [62]. In another study [63], we showed via biophysical modeling of OS stability, that changes in protein composition in OS discs may lead to breakage of the OS. If the OS is the site of toxicity, enhancing the folding and transport of Rho^P23H^ to the OS may be detrimental to the cell. Since haploinsufficiency does not cause severe degeneration [60], [61] and Rho^P23H^ is preferentially degraded over wild type Rho [11], investigating the effects of enhancing the degradation of Rho^P23H^ may determine if decreasing the transport of Rho^P23H^ to the OS prevents the disorganization/displacement of OS discs and protects against photoreceptor death. Therefore, the role of the OS and protein degradation mechanisms in RP need to be investigated in the future.

## Supporting Information

Figure S1 **Representative ERG waveforms from** ***GHL^+^*** **and** ***GHL^+^/Chop^−/−^*** **mice.** Average scotopic ERG a- and b-wave waveforms from (A) 4 and 28 week old C57BL/6 (blue traces), *Chop^−/−^* (orange traces) (B) 4–28 week old *GHL^+^* (black traces) and *GHL^+^/Chop^−/−^* (red traces) mice in response to 1 ms flashes of increasing intensity, from bottom to top, (*n = 6–14*). Error bars ± SD. Scale bar: x = 50 ms, y = 200 µV. Arrows represent onset of light stimulus.
(EPS)

Figure S2 **Representative ERG waveforms from** ***GHL^+^*** **and** ***GHL^+^/Ask1^−/−^*** **mice.** Average scotopic ERG a- and b-wave waveforms from (A) 4 and 28 week old C57BL/6 (blue traces), *Ask1^−/−^* (green traces) (B) 4, 16, and 28 week old *GHL^+^* (black traces) and *GHL^+^/Ask1^−/−^* (purple traces) mice in response to 1 ms flashes of increasing intensity, from bottom to top, (*n = 7–14*). Error bars ± SD. Scale bar: x = 50 ms, y = 200 µV. Arrows represent onset of light stimulus.
(EPS)
